# Notícias de uma guerra oitocentista

**DOI:** 10.1590/S0104-59702024000100014

**Published:** 2024-04-15

**Authors:** Márcio Magalhães de Andrade

**Affiliations:** i Doutor em História das Ciências e da Saúde/Fiocruz. Rio de Janeiro – RJ – Brasil marcmagand@gmail.com

Apesar de ter nascido em São Cristóvão, bairro carioca repleto de referências à Guerra do Paraguai, sempre tive a certeza de que meu conhecimento sobre o conflito sul-americano era escasso e equivocado em vários aspectos. Não me diziam muita coisa os nomes de ruas e lugares como Curuzu, Tuiuti, Carneiro de Campos, Coronel Cabrita, General Bruce, General Argolo e Figueira de Melo, topônimos de referência em minhas andanças nos idos de infância e adolescência. Lima Barros, rua onde cresci, era nada mais nada menos que uma homenagem a Francisco José de Lima Barros, jovem guarda-marinha e um dos muito combatentes mortos no dia 11 de junho de 1865, na famosa batalha naval do Riachuelo. Pouco depois viria a ser também o nome de um dos encouraçados brasileiros usados no mesmo conflito.

O livro publicado pelo historiador Leo Bahiense (2022) constitui um excelente convite àqueles que ainda não se dedicaram de forma mais demorada ao assunto. Em *Doutores, enfermos e canhões: uma história médica da Guerra do Paraguai* (1864-1870), o autor demonstra considerável fôlego e erudição, costurando informações oriundas de muitas fontes primárias e de vasta bibliografia. A empreitada de Bahiense dá ao seu trabalho o *status* de obra de referência por três motivos, essencialmente: (1) reúne informações de variadas origens (memórias de veteranos, teses médicas, bibliografia sobre medicina e guerras, sobretudo mas não somente as do século XIX); (2) analisa aspectos pouco ou nada abordados por outros autores que se dedicaram à Guerra da Tríplice Aliança, evidenciando características e dilemas da medicina oitocentista e das relações, muitas delas conflituosas, de pessoas de carne e osso, simples mortais; (3) compara, pela perspectiva da medicina, a Guerra do Paraguai a outros conflitos no globo (guerras napoleônicas, Guerra de Secessão, Guerra da Crimeia, além das duas grandes guerras mundiais).

Concordo com a observação feita pelo professor Luiz Otávio Ferreira (2022) no prefácio do livro de Bahiense, quando afirma tratar-se de um trabalho de antropologia histórica, que busca a compreensão da guerra como um fenômeno cultural de larga amplitude, e não somente como resultado de questões geopolíticas e de intervenções militares. O livro em questão opta pelo aprofundamento e pela densidade ao fazer uso de um conjunto de narrativas sincrônicas, e não diacrônicas, como é padrão nos trabalhos de cunho histórico. Nesse aspecto, *Doutores, enfermos e canhões* se aproxima da abordagem de Richard Hollingham (2011), que, em *Sangue e entranhas: a assustadora história da cirurgia*, evita a cronologia e organiza seus capítulos de forma temática ao abordar as experiências cirúrgicas ao longo da história.

Quais eram os impasses da medicina e das práticas médicas eruditas e populares no distante Oitocentos, quando um hegemônico e difuso neo-hipocratismo tinha que dar conta de um mundo de sofrimentos bem distintos dos nossos (já reinterpretados à luz da bacteriologia e de outras áreas de conhecimento mais recentes na história das ciências)? Quais foram os tipos de hospitais acessíveis aos combatentes durante a Guerra do Paraguai? Quais eram os debates sobre técnicas para cirurgias e amputações? Que tipo de armas e projéteis foram utilizados durante o conflito? E os instrumentos para a retirada de objetos que invadiam e estraçalhavam carne humana? Além de estampidos, pólvora, fogo e lâminas, como deve ter sido sobreviver e morrer em decorrência das intempéries, das carências nutricionais, do cansaço, dos pântanos, alagadiços e ares corruptos que pareciam dar total sentido à ocorrência de surtos de cólera, varíola, beribéri, malária, disenteria, tétano, gangrena e escorbuto? Parte desse inferno existencial consta nas páginas de Francisco Doratioto (2002, p.127), outra referência incontornável sobre a Guerra do Paraguai:

Sem recursos logísticos e sem força militar suficiente, o coronel Camisão teve que recuar em sua decisão de alcançar Concepción. Ordenou, em 7 de maio de 1867, a retirada para Nioaque, que ficou conhecida como a Retirada da Laguna, e à qual se incorporaram índios Guaicuru e Terena. A retirada foi feita sob constantes ataques dos paraguaios, que arrebataram à coluna o gado de corte, o que a levou, novamente, à fome. Os soldados brasileiros marcharam, famintos, sob incessantes tempestades e por terreno pantanoso; tinham a incomodá-los, além dos inimigos, piolhos, e a vitimá-los o cólera e outros problemas de saúde, decorrentes do contraste entre o frio glacial noturno e o calor escaldante diurno. Para encurralar os retirantes, as forças paraguaias ateavam fogo no mato, alto e seco, que os asfixiava e os instava à rendição, sempre recusada.

Aquelas foram algumas das muitas perguntas formuladas e respondidas por Leo Bahiense, que se preocupou em dar concretude e provocar efeitos de verdade (*enargeia*) ao narrar o sofrimento alheio, ao falar sobre personagens nada conhecidos na história da Guerra da Tríplice Aliança e ao descrever as entranhas expostas pela barbárie de todo e qualquer conflito daquela natureza. Graças à opção metodológica e teórica do autor, conseguimos saber algo sobre personagens como Júlio José das Chagas, José Rodrigues de Campos, Paulino Ovídio Barbosa, José Antônio dos Santos Cariman, João Fernandes Eiras, “Soldado A”, “Soldado B”, “Soldado C”, “Soldado D”, entre outros. Afinal, nem só de militares de alta patente, médicos e cirurgiões foi feita a Guerra do Paraguai.

Seguindo essa linha de argumentação e espraiando os limites geográficos e temporais, Bahiense evidencia e nos provoca sobre a necessidade de pesquisas sobre o papel dos esquecidos da história, sobre mulheres e homens de várias cores, mestiços e negro-mestiços que carregaram no corpo as nefastas consequências da vil e infamante escravidão.

**Figura 1:**
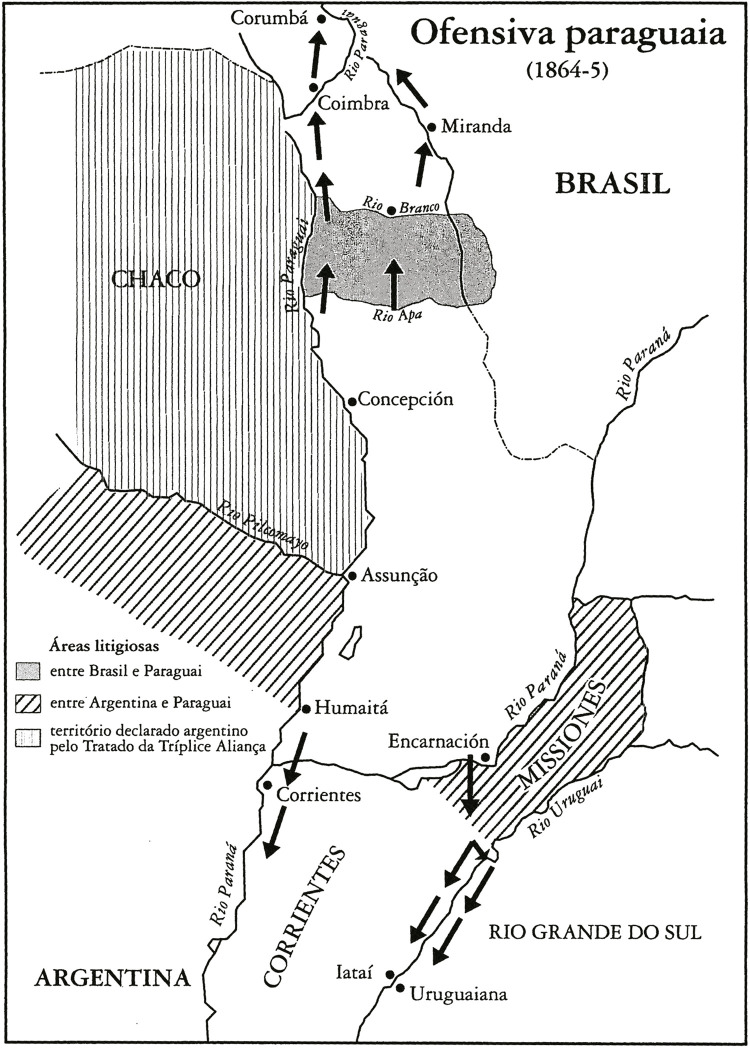
: Mapa da ofensiva paraguaia entre 1864 e 1865 (Doratioto, 2002, p.89)

Quando da Guerra do Paraguai, muitos escravos aceitariam, como facultado por lei, fazer a Guerra no lugar de seu senhor, ou dos filhos de seus senhores, em troca da alforria imediata, das vantagens já especificadas e da perspectiva de carreira militar. O Decreto n.2.725, de 6 de novembro de 1865, libertava os chamados ‘escravos da nação’ que quisessem partir para o serviço de guerra, como muitos fizeram. ... Muitos escravos, libertos e homens livres, da mesma forma, tomaram o alistamento como prova de bravura pessoal e via de integração na sociedade mais ampla (Silva, 1995, p.71).

E mais: que contribuições à história da humanidade deram mulheres como Florence Nightingale, Dorothea Lynde Dix e Ana Néri? No desenterrar de histórias ocultadas por interpretações machistas, além das racistas, algumas páginas são dedicadas à irmã Paula, por exemplo.

Insisto na dimensão convidativa do trabalho em foco. Tal dimensão é válida, inclusive, para as eventuais divergências. Senti-me provocado e tentado a estudar mais sobre o intelectual português Boaventura de Sousa Santos (2014, 2018) e suas “epistemologias do Sul”. Em *Doutores, enfermos e canhões*, o “Sul epistemológico” ajudaria, por exemplo, na identificação de protagonismos políticos e intelectuais dos subalternos. Nesse aspecto, as referências a Boaventura pareceram ter muito mais a ver com uma manifestação de intenção de Bahiense do que um eixo para sua análise. As fontes e alguns deslocamentos de olhar lhe permitiram personagens novos, pouco usuais e discriminados por uma “história oficial” e “vista de cima”, mas é claro que têm seus limites. As perguntas do historiador, por mais criativas e engenhosas que sejam, não podem nem devem alterar fatos. Leo Bahiense sabe muito bem e manifesta sua atenção em algumas passagens: “Na verdade, a militância política – indissociável do apaixonamento – pode ser uma companhia perigosa para o historiador”.

Sejamos apaixonados, sensíveis aos dilemas e injustiças de nosso tempo, mas nunca percamos de vista a seriedade e o profissionalismo no ofício do historiador.

Por último, mas não menos importante: o recurso a mapas e a um índice remissivo deixaria o trabalho de Leo Bahiense (e de outros historiadores) ainda mais atraente e compreensível, não só para o público especializado, mas principalmente para o público leigo amante das boas histórias.
